# Proteomic Mediators Linking Autoimmune Diseases to Major Adverse Cardiovascular Events: Insights from the UK Biobank

**DOI:** 10.3390/proteomes14030038

**Published:** 2026-07-24

**Authors:** Jingwen Huang, Chang Liu, Laurence S. Sperling, Arshed A. Quyyumi, Yan V. Sun

**Affiliations:** 1 Division of Cardiology, Department of Medicine, Emory University School of Medicine, Atlanta, GA 30322, USA; 2 Department of Epidemiology, Emory University Rollins School of Public Health, Atlanta, GA 30322, USA; 3 Veterans Affairs Atlanta Healthcare System, Decatur, GA 30033, USA

**Keywords:** major adverse cardiovascular events, cardiovascular death, autoimmune disease, proteomics, mediation analysis, PWAS

## Abstract

Background: Autoimmune diseases (AIDs) are associated with increased cardiovascular risk. However, specific protein mediators linking AIDs to major adverse cardiovascular events (MACE) and cardiovascular death (CV death) remain unexplored. This study identifies proteomic mediators linking AIDs to MACE via high-dimensional mediation analysis in the UK Biobank. Methods: We used UK Biobank data with proteomic profiling by Olink platform. Participants with prevalent myocardial infarction (MI), stroke, and heart failure at baseline were excluded. AIDs were categorized into musculoskeletal (MSK), vasculitis, gastrointestinal (GI), neurologic, and rheumatic fever subsets. Fine–Gray models assessed associations between AIDs and MACE and CV death. Proteome-wide association studies identified proteins associated with both AIDs and cardiovascular outcomes. High-dimensional mediation analysis (HIMA) explored protein-mediated pathways. All models adjusted for age, sex, lipids, BMI, smoking, hypertension, diabetes, chronic kidney disease, atrial fibrillation, and coronary artery disease. Results: Among 400,633 participants (median follow-up 14.5 years, 44.8% male), AIDs were present in 28,754 (7.2%). All AID categories were associated with increased MACE (sHR: MSK 1.34, vasculitis 1.67, GI 1.20, neurologic 1.33, rheumatic fever 1.38; all *p* < 0.001). For CV death, MSK, vasculitis, and rheumatic fever showed increased risk (sHR 1.34, 1.78, 1.51; all *p* ≤ 0.004), but not GI or neurologic AIDs. In 43,599 participants with proteomic data, HIMA identified 66 and 32 unique potential mediators linking AIDs to MACE and CV death, respectively. Four proteins (Growth Differentiation Factor 15, Interleukin-15, urokinase plasminogen activator receptor, and Tenascin C) mediated the AID-MACE relationship across multiple AID categories. Growth Differentiation Factor 15 and Interleukin-15 were shared mediators for CV death. Conclusions: This proteomic analysis identifies specific proteins that may mediate the association between AIDs and adverse cardiovascular outcomes, offering mechanistic insights into immune-related cardiovascular risk. These findings are hypothesis-generating and require replication and validation before the identified proteins can be considered causal mediators or adopted for clinical risk stratification.

## 1. Introduction

Cardiovascular disease (CVD) remains the leading cause of morbidity and mortality worldwide, responsible for a significant number of deaths and disabilities [1]. Major adverse cardiovascular events (MACE) (including MI, stroke, heart failure [HF], and cardiovascular death [CV death]) represent critical manifestations of advanced cardiovascular disease with substantial impacts on patient outcomes and healthcare systems [2,3]. While traditional risk factors such as hypertension, diabetes, and dyslipidemia are well established, emerging evidence suggests that systemic inflammation plays a crucial role in cardiovascular pathogenesis through endothelial dysfunction, atherosclerotic plaque formation, and thrombotic complications [4,5].

Autoimmune diseases (AIDs) represent a diverse group of conditions characterized by immune-mediated tissue damage and chronic systemic inflammation [6]. Recent epidemiological studies have demonstrated associations between various AIDs and increased cardiovascular risk [7,8]. Patients with rheumatoid arthritis exhibit a 50–70% increased risk of MI compared to the general population [9]. Similarly, systemic lupus erythematosus has been associated with accelerated atherosclerosis and premature CVD, with a 2–3-fold increased risk of cardiovascular events [10]. However, the molecular mechanisms underlying these associations remain incompletely understood. AIDs confer a 1.5–3.5-fold increased cardiovascular risk independent of traditional risk factors, as demonstrated across 22 million UK individuals [11]. Chronic cytokine excess (IL-6, TNF-α, IL-1β) drives endothelial dysfunction, pro-thrombotic states, and accelerated atherosclerosis [12], while Olink-based proteomic studies have identified broad cardiovascular protein dysregulation in AID patients and demonstrated that circulating proteins improve MACE prediction in the general population [13]. However, the specific proteins mediating the AID-MACE relationship across disease categories remain uncharacterized.

The circulating proteome represents a rich source of biomarkers that may reveal pathophysiological mechanisms linking AIDs to cardiovascular outcomes. Recent advances in high-throughput proteomics enable comprehensive assessment of plasma proteins that may serve as mediators in disease pathways [14]. Studies have identified inflammatory proteins such as C-reactive protein, interleukin-6, and tumor necrosis factor-alpha as associated with CVD [5,15,16] but systematic evaluation of protein mediators in the context of autoimmune-related CVD has not been performed.

In this study, we leveraged the UK Biobank, a large-scale prospective cohort with deep phenotyping and proteomic profiling, to identify protein mediators linking AIDs to MACE and CV death. Identification of mediating pathways could potentially provide novel therapeutic targets and inform risk stratification strategies beyond standard CVD prevention in patients with autoimmune conditions.

## 2. Materials and Methods

### 2.1. Study Population

The UK Biobank enrolled approximately 500,000 participants aged 40–69 years across the United Kingdom between 2006 and 2010 in this population-based prospective cohort study. Detailed study design and recruitment procedures have been previously described [17]. The Northwest Multi-Center Research Ethics Committee approved the study (reference number 16/NW/0274), with all participants providing written informed consent. At baseline, participants completed comprehensive assessments encompassing questionnaires, physical examinations, and biological specimen collection. Longitudinal linkage to national health records provides ongoing data on inpatient admissions through Hospital Episode Statistics, primary care encounters, mortality events via national death registries, and participant-reported medical conditions. All diagnostic and mortality data were standardized using International Classification of Diseases, Tenth Revision (ICD-10) coding [11,18].

AIDs were stratified into five categories according to predominant organ system affected: musculoskeletal (MSK) AIDs encompassing rheumatoid arthritis, enteropathic arthropathies, systemic lupus erythematosus, systemic sclerosis, Sjögren syndrome, systemic connective tissue disorders, ankylosing spondylitis, psoriasis, and psoriatic arthropathies; vasculitis encompassing polyarteritis nodosa and necrotizing vasculopathies; gastrointestinal (GI) AID encompassing Crohn disease, ulcerative colitis, celiac disease, and inflammatory liver disease; neurologic AID encompassing multiple sclerosis, acute disseminated demyelination, myasthenia gravis, and inflammatory polyneuropathy; and rheumatic fever excluding cardiac involvement. To establish appropriate temporal relationships, only AIDs present at baseline were designated as exposures. Complete ICD-10 coding algorithms for AID identification and cardiovascular outcome ascertainment are provided in Table A1.

Disease prevalence and incidence were established by comparing first onset dates for AIDs and cardiovascular outcomes relative to UK Biobank enrollment, with diagnoses preceding enrollment designated as prevalent and those occurring post-enrollment designated as incident. The analytical cohort excluded participants with prevalent MI, HF, or stroke at baseline. Follow-up duration for incident events spanned from enrollment through the earliest occurrence of outcome event, loss to follow-up, death, or end of follow-up in March 2023. CV death was defined as mortality attributed to cardiovascular causes, including acute MI, sudden cardiac death, HF, stroke, or other disease of the heart and vasculature, based on ICD-10 coding of the primary and secondary causes (Table A1) of death on death-records. CV death constituted a distinct endpoint for independent mortality assessment.

**Figure 1:**
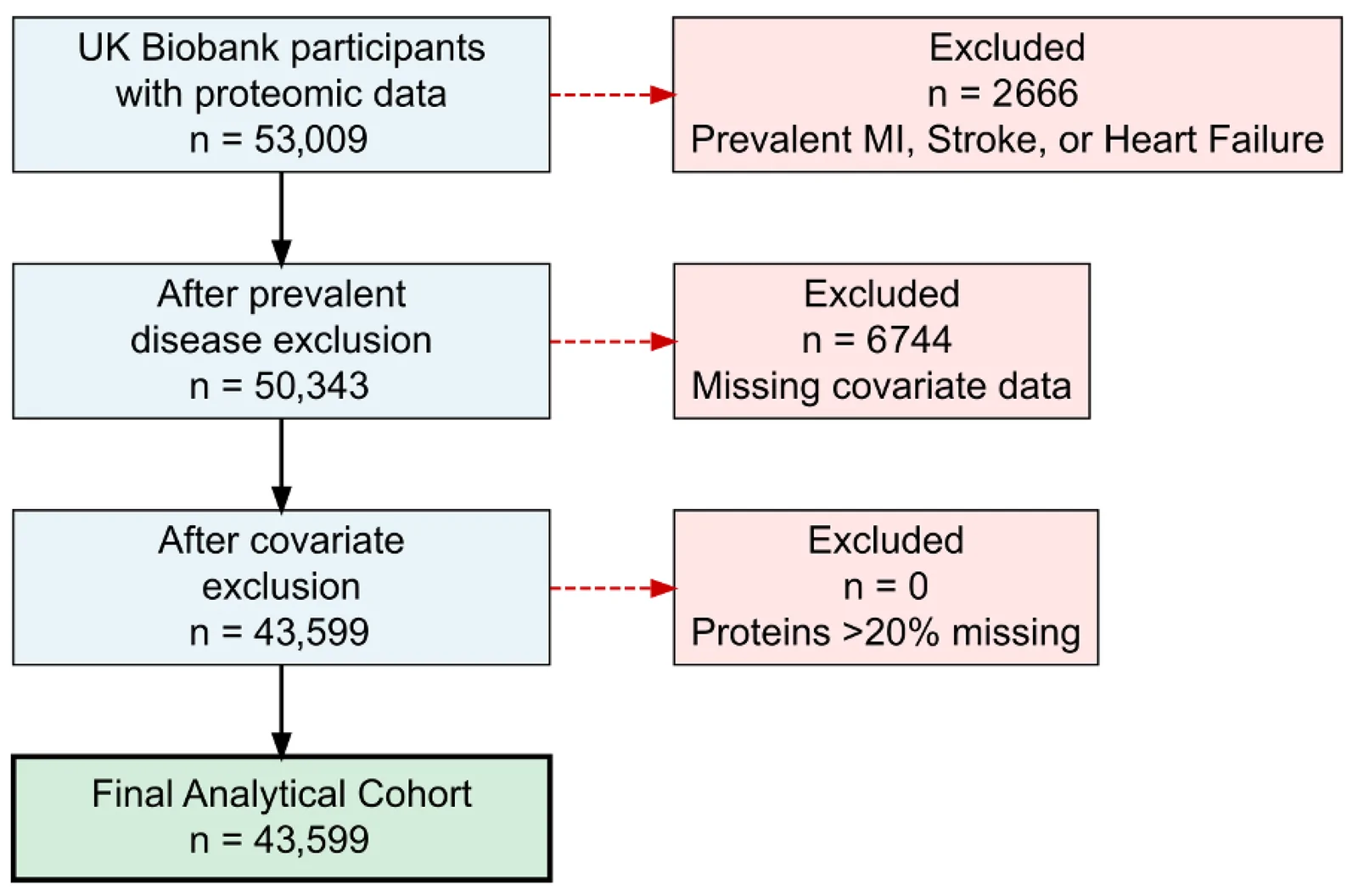
Study cohort selection flowchart for proteomic analysis. Participant selection from UK Biobank proteomic study for major adverse cardiovascular event and cardiovascular death analysis.

Proteomic data were generated by the UK Biobank Pharma Proteomics Project (UKB-PPP), which selected 53,009 participants using a two-component design: a randomly selected subset broadly representative of the full UK Biobank, and a consortium-selected subset enriched for specific disease phenotypes [19]. Plasma proteomic profiling utilized the Olink Explore 3072 proximity extension assay (PEA) platform (Olink Proteomics, Uppsala, Sweden), quantifying 2923 distinct protein targets [19]. Measurements were reported as normalized protein expression (NPX) values on a log2 scale. Quality assurance protocols excluded proteins exceeding 20% missing data and samples not meeting platform-specified quality standards. To optimize statistical properties and minimize outlier influence, protein levels underwent rank-based inverse normal transformation. The proteomic analytical cohort comprised participants free of prevalent MI, HF, or stroke with available proteomic measurements. The primary sources of missingness were HDL cholesterol (12.8%), LDL cholesterol (5.1%), and BMI (0.4%), while age, sex, smoking status, hypertension, type 2 diabetes, prevalent CAD, and prevalent atrial fibrillation had no missing values. Application of complete-case analysis yielded a final analytical sample of 43,599 participants. (Figure 1).

### 2.2. Statistical Analysis

#### 2.2.1. Association of AIDs with Cardiovascular Outcomes

The primary outcome was MACE, which included MI, stroke, HF, and CV death. In secondary analysis, CV death was examined separately as a distinct endpoint representing the most severe manifestation of cardiovascular disease. To evaluate the relationship between each AID category and cardiovascular outcomes (MACE in primary analysis and CV death in secondary analysis), we employed Fine–Gray subdistribution hazard models while treating non-CV death as a competing risk [20]. Adjustments were made for age, sex, body mass index (BMI), smoking history, high-density lipoprotein (HDL) cholesterol, low-density lipoprotein (LDL) cholesterol, and history of hypertension, type 2 diabetes mellitus, chronic kidney disease, atrial fibrillation, and coronary artery disease. Findings were reported as subdistribution hazard ratios (sHR) along with 95% confidence intervals (CI) and *p*-values.

#### 2.2.2. Proteome-Wide Association Studies

Separate proteome-wide association studies (PWAS) were performed for each AID and cardiovascular outcome. Initially, we utilized linear regression to determine which proteins were associated with each AID category, using rank-based inverse normal transformed protein levels as dependent variables and AID status as the independent variable, with adjustment for age, sex, BMI, smoking status, HDL, LDL, and comorbidities as previously outlined. Subsequently, Fine–Gray models were applied to determine proteins associated with MACE and CV death, using protein levels as independent variables while accounting for non-CV death as a competing risk, applying identical covariate adjustments. Bonferroni correction for multiple testing established statistical significance at *p* < 1.71 × 10^−5^, calculated by dividing the *p*-value threshold of 0.05 by the total number of proteins tested (0.05/2923).

#### 2.2.3. High-Dimensional Mediation Analysis

For the proteins that showed significant associations with both AID and cardiovascular outcome in PWAS, we conducted high-dimensional mediation analysis (HIMA) to evaluate whether these proteins serve as mediators in the pathway between AID and adverse cardiovascular outcomes. HIMA utilizes a two-phase methodology: (1) identifying potential mediators through sure independence screening, and (2) performing joint significance testing with de-biased lasso regression to simultaneously account for multiple mediators [21,22]. This approach detects mediators exhibiting non-zero indirect effects while maintaining control over the family-wise error rate. Because HIMA framework does not support subdistribution hazards for competing risks, the mediation step used standard Cox proportional hazards models for the protein-to-outcome association, censoring for non-cardiovascular death. Adjustments in all mediation analyses were as outlined above. HIMA calculates two critical parameters for each protein: α (beta coefficient, representing the influence of AID on protein levels) and exp (β) (hazard ratio, representing the influence of protein levels on cardiovascular outcome risk). The indirect effect (IDE hazard ratio exp (α × β)) measures the portion of AID’s effect on cardiovascular outcomes transmitted through each protein mediator. Significance was established using the higher *p*-value obtained from testing both the AID-to-protein effect and the protein-to-cardiovascular outcome effect, with mediation deemed significant when the joint *p*-value < 0.05. As candidate proteins entering HIMA were already required to survive Bonferroni correction at both the AID-protein and protein-outcome PWAS steps, the HIMA joint *p*-value threshold of 0.05 was applied to a pre-filtered, high-confidence candidate at this stage. Mediators were prioritized according to clinical relevance by computing relative importance, defined as each mediator’s proportional contribution to the overall mediated effect (Graphic Abstract). Statistical analyses were conducted using R version 4.5.1. Unless specified otherwise for PWAS and HIMA, a two-sided *p*-value < 0.05 indicated statistical significance.

To draw causal conclusions from mediation analysis, structural assumptions about the absence of confounding must hold. Causal mediation inference requires four such assumptions (C.1–C.4) as enumerated in Zhang et al. [22]: no unmeasured confounders of the (C.1) AID-outcome, (C.2) protein-outcome, or (C.3) AID-protein relationships, and (C.4) no exposure-induced confounders of the protein-outcome path. C.1 and C.3 are partially supported by our covariate adjustment strategy, though residual confounding from physical inactivity, socioeconomic deprivation, and subclinical inflammation cannot be excluded. C.2 is the most vulnerable assumption, as immunosuppressive and biological therapies used in AID patients—not captured in UK Biobank data—directly modulate protein levels and cardiovascular risk. C.4 cannot be verified, as AID-related organ complications may simultaneously alter protein levels and cardiovascular risk, creating exposure-induced confounders that cannot be adjusted for without introducing collider bias. These limitations preclude definitive causal interpretation; findings should be regarded as evidence of statistical mediation rather than confirmed causal pathways.

## 3. Results

### 3.1. Baseline Characteristics

The study included 400,633 UK Biobank participants without prevalent MI, HF, or stroke at baseline (Table 1(A)). The median enrollment age was 57 years (IQR 50–63) and 44.8% were male. Among the participants, 28,754 participants (7.2%) had prevalent AID at baseline: 16,568 (4.1%) with MSK AID, 680 (0.2%) with vasculitis, 8348 (2.1%) with GI AID, 1900 (0.5%) with neurologic AID, and 1258 (0.3%) with rheumatic fever. Participants with AIDs were slightly older, more likely to be females, and had higher prevalence of traditional cardiovascular risk factors. Median follow-up time was 14.5 years (IQR 13.6–15.2). A total of 43,599 participants had proteomic data available for PWAS and mediation analysis (Table 1(B)); the full-cohort analysis (N = 400,633) was conducted to establish the epidemiological context for the AID-MACE association, providing the rationale for the subsequent proteomic mediation analysis.

### 3.2. Association Between Autoimmune Diseases and Cardiovascular Outcomes

During follow-up, substantial numbers of participants developed adverse cardiovascular outcomes across AID categories. In the primary analysis of the full cohort (Table 2(A)), MSK was associated with significantly higher risk of MACE (sHR 1.34, *p* < 0.001) compared to individuals with no AID. Vasculitis demonstrated the strongest association with MACE (sHR 1.67, *p* < 0.001), followed by rheumatic fever (sHR 1.38, *p* < 0.001), neurologic AID (sHR 1.33, *p* < 0.001), and GI AID (sHR 1.20, *p* < 0.001). For CV death, vasculitis (sHR 1.78, *p* < 0.001) and rheumatic fever (sHR 1.51, *p* = 0.004) showed the strongest associations, while GI and neurologic AID were not significantly associated.

These findings were largely consistent in the proteomic sub-cohort (Table 2(B)). MSK remained significantly associated with both MACE (sHR 1.38, *p* < 0.001) and CV death (sHR 1.41, *p* = 0.004). Vasculitis again demonstrated the strongest associations with MACE (sHR 1.71, *p* = 0.002) and CV death (sHR 1.98, *p* = 0.02), though with wider confidence intervals reflecting the smaller sample. GI AID showed a modest association with MACE (sHR 1.21, *p* = 0.04) but not CV death. Rheumatic fever and neurologic AID did not reach statistical significance in the sub-cohort, likely reflecting reduced power given their low prevalence (0.3% and 0.8%, respectively).

### 3.3. Proteome-Wide Association Studies

**Figure 2:**
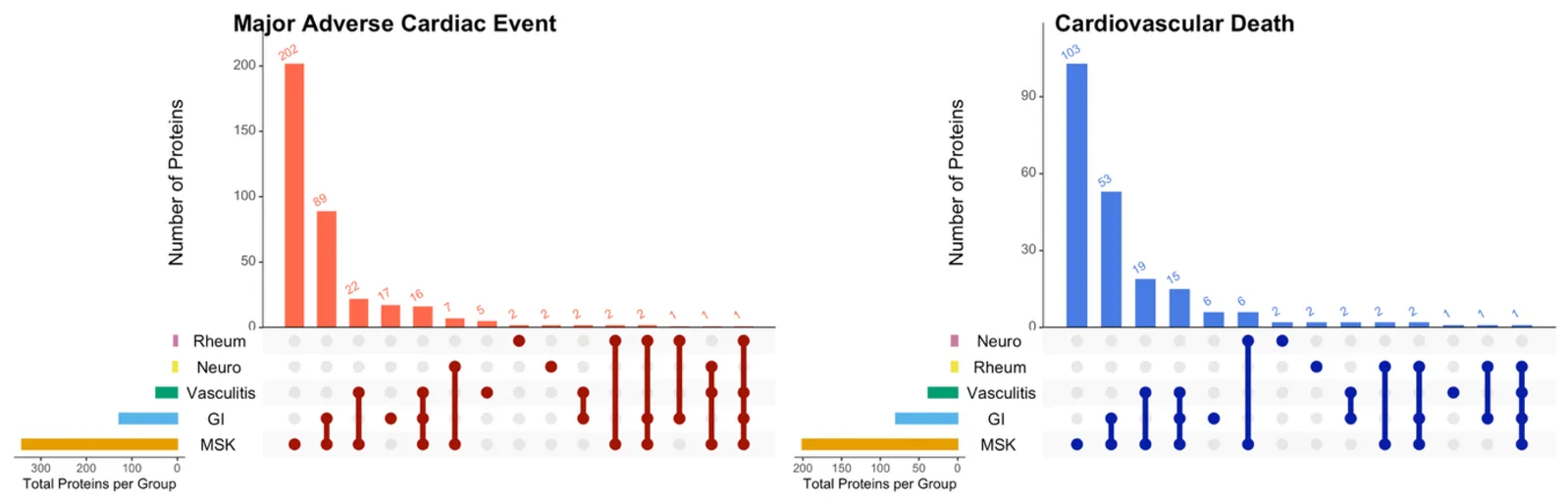
Overlapping proteomic profiles linking autoimmune diseases to adverse cardiovascular outcomes. UpSet plots showing the number and overlap of proteins associated with both autoimmune disease categories and adverse cardiovascular outcomes that were advanced to high-dimensional mediation analysis. Left panel: proteins associated with major adverse cardiovascular events (MACE, composite of myocardial infarction, stroke, heart failure, and cardiovascular death); Right panel: proteins associated with cardiovascular death. Bar charts (top) display the number of proteins for each combination, with connected dots (bottom) indicating which autoimmune disease categories share proteins. MSK: musculoskeletal autoimmune diseases; GI: gastrointestinal autoimmune diseases; Neuro: neurologic autoimmune diseases; Rheum: rheumatic fever; Vasculitis: systemic vasculitis. Left bar charts show the total number of proteins associated with each autoimmune disease category.

The PWAS identified varying numbers of proteins associated with each AID category: MSK AID (475 proteins), GI AID (158 proteins), vasculitis (53 proteins), rheumatic fever (12 proteins), and neurologic AID (22 proteins). For cardiovascular outcomes, 576 and 284 proteins were significantly associated with MACE and CV death, respectively. Cross-referencing these results identified proteins associated with both AIDs and cardiovascular outcomes, which were advanced to HIMA (Figure 2).

### 3.4. Protein Mediators Linking Autoimmune Diseases to Cardiovascular Oucomes

**Figure 3:**
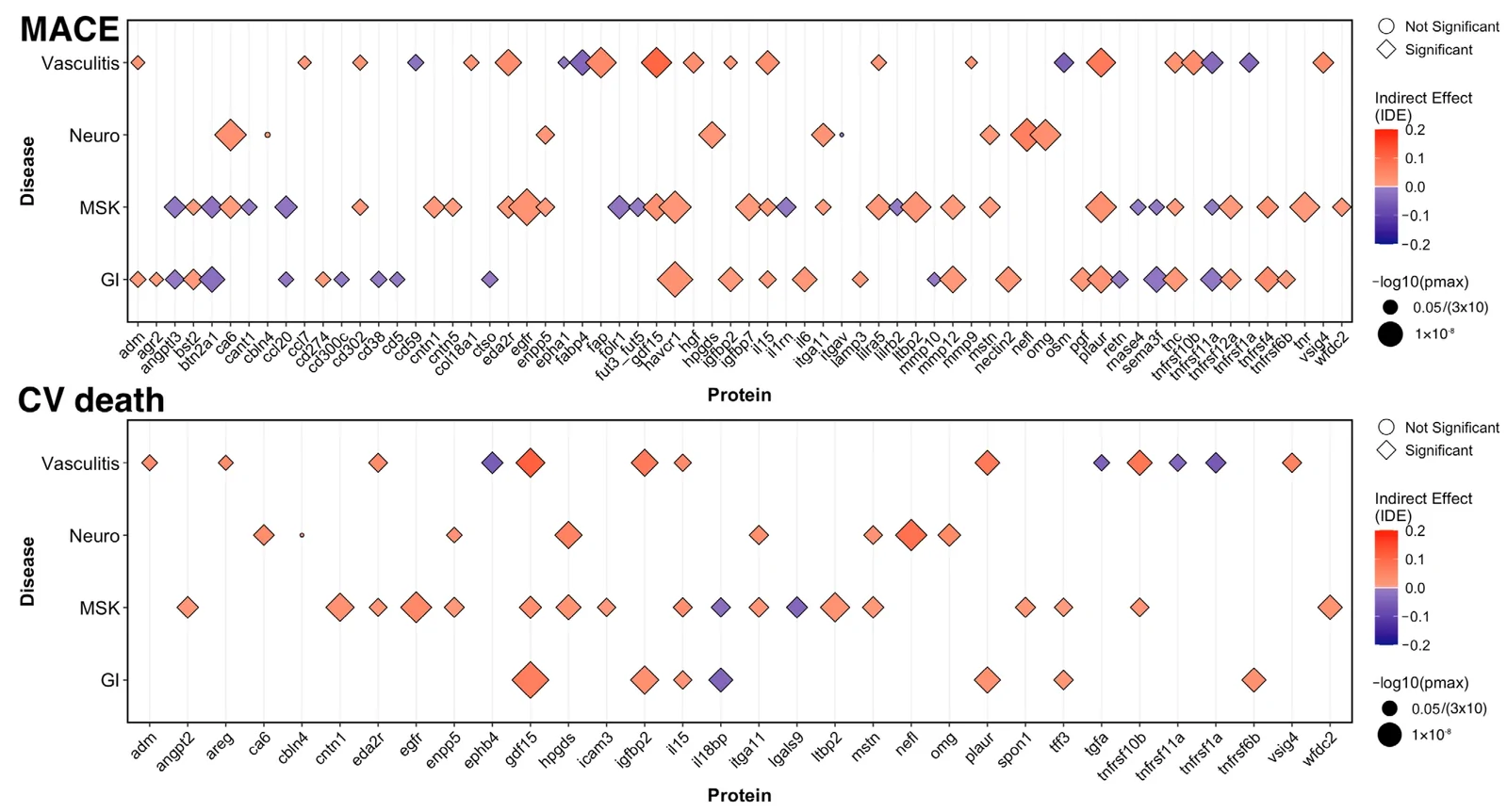
Protein mediators linking autoimmune diseases to cardiovascular outcomes. The bubble plot shows significant protein mediators identified through High-Dimensional Mediation Analysis (HIMA) linking autoimmune disease exposures (musculoskeletal [MSK], vasculitis, gastrointestinal [GI], and neurologic) to cardiovascular outcomes (MACE and CV death) in UK Biobank participants with proteomic data. Pmax represents the joint *p*-value, which is defined as the larger of the *p*-values for the AID-protein and protein-cardiovascular outcome paths. The indirect effect (IDE = effect of AID on protein × effect of protein on cardiovascular outcomes) is the product of two beta coefficients and represents the mediation coefficient estimates from AID to cardiovascular outcomes, adjusted for age, sex, HDL, LDL, BMI, history of smoking, hypertension, type 2 diabetes, chronic kidney disease, prevalence of coronary artery disease, and prevalence of atrial fibrillation. The color intensity indicates the magnitude of mediation effect, while color itself indicates the direction (red = risk-increasing, blue = lower-risk). MACE: major adverse cardiac effect; CV death: cardiovascular death; BMI: body mass index; HDL: high-density lipoprotein cholesterol; LDL: low-density lipoprotein cholesterol.

**Figure 4:**
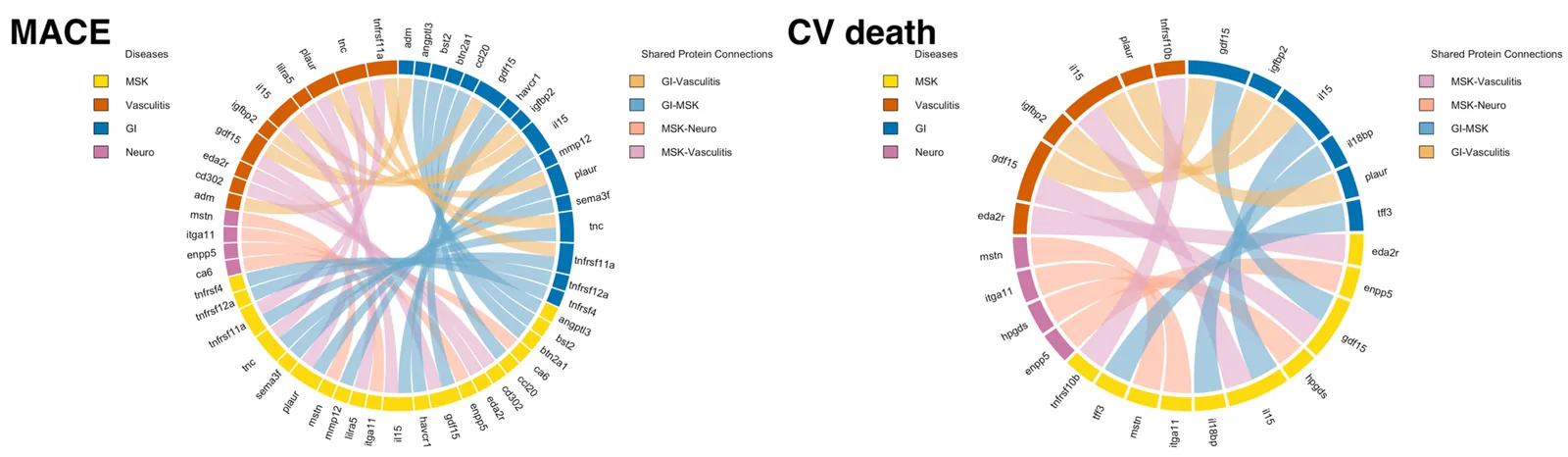
Shared protein mediators across various autoimmune diseases by outcomes. The Circos plot displays proteins shared across multiple autoimmune disease groups by cardiovascular outcomes-musculoskeletal [MSK], vasculitis, gastrointestinal [GI], and neurologic autoimmune diseases.

HIMA identified substantial numbers of protein mediators linking AIDs to adverse cardiovascular outcomes, with both shared mediators across outcomes and outcome-specific signatures (Figure 3). In the primary analysis, a total of 66 proteins statistically mediated the relationship between AIDs and MACE. In the secondary analysis, 32 proteins mediated the relationship with CV death. Of note, no significant mediators were identified for rheumatic fever category. Detailed results for MACE, and CV death are shown in Table A2 and Table A3, respectively. Shared proteins between AID groups for each cardiovascular outcome are shown in Figure 4.

Primary Analysis: Protein mediators of MACE. For MACE, four proteins were shared across MSK, GI, and vasculitis, of which growth differentiation factor 15 (GDF15), interleukin-15 (IL-15), soluble urokinase plasminogen activator receptor (PLAUR/suPAR), and Tenascin C (TNC) are the most prominent universal mediators, representing universal mediators of immune-related cardiovascular pathogenesis (Figure 3, Table A2). GDF15 demonstrated the strongest and most consistent mediation effects across these three AID categories, with relative importance ranging from 5.9% to 14.0%, while PLAUR/suPAR showed consistent strong mediation (relative importance 6.1% to 9.3%) as well. AID-category-specific mediators included Interleukin-6 (IL-6) and HAVCR1/KIM-1in GI AIDs; hepatocyte growth factor (HGF) and oncostatin M (OSM) in vasculitis; and neurofilament light chain (NEFL) and oligodendrocyte myelin glycoprotein (OMG) in neurologic AIDs. The vasculitis-specific mediator findings rest on a modest analytic base, and while the direction of associations was consistent with the full-cohort analysis, the wide confidence intervals preclude definitive conclusions. These results should be interpreted as hypothesis-generating, and larger studies dedicated to vasculitis populations are needed to confirm or refute the identified mediators.

Secondary Analysis: For CV death, GDF15 and IL-15 were shared mediators across the same three AID categories—MSK, GI, and vasculitis (Figure 3, Table A3). GDF15 again showed the most robust mediation effects, with relative importance ranging relative importance 8.3% to 27.5%, whereas IL-15 demonstrated consistent but more modest mediation effects across these categories (5.6 to 9.8%). CV death-specific mediators included Interleukin-18 binding protein (IL18BP, relative importance 6.7% to 17.8%) and latent transforming growth factor beta binding protein 2 (LTBP2, relative importance 6.0% in MSK). Neurologic-related proteins NEFL and OMG emerged prominently in neurologic AIDs with NEFL showing the highest relative importance (27.9%) for CV death in this AID category.

Gene Ontology-based enrichment analysis of the HIMA-selected mediators identified consistent enrichment of cell adhesion, inflammatory signaling, and vascular remodeling pathways across AID categories for both MACE and CV death (Table A4 and Table A5).

## 4. Discussion

This large-scale proteomics study provides comprehensive mechanistic insights into the relationship between AIDs and adverse cardiovascular outcomes by identifying protein mediators linking AIDs to MACE and CV death. Our findings demonstrate that AIDs significantly increase the risk of adverse cardiovascular outcomes. Through HIMA, we identified numerous protein mediators that at least partly mediated this risk, providing evidence for common and distinct pathophysiological mechanisms underlying different manifestations of CVD in autoimmune conditions.

### 4.1. Shared Mediators Across Cardiovascular Outcomes

GDF15 is a stress-responsive cytokine induced by oxidative stress, inflammation, and tissue injury [23]. In cardiovascular disease, elevated GDF15 reflects ongoing tissue injury, metabolic stress, and inflammatory burden [24,25]. GDF15 has been strongly associated with adverse cardiovascular events and cardiovascular mortality across diverse populations [26,27,28]. Our findings demonstrate that GDF15 statistically mediates the relationship between three major AID categories, suggesting that GDF15 serves as a unified biomarker reflecting the cumulative cardiovascular burden of chronic inflammation across diverse autoimmune conditions, making it a prime candidate for risk stratification in AID patients.

IL-15 is a pro-inflammatory cytokine that promotes T-cell and NK-cell proliferation and activation [29,30]. IL-15 is a pro-inflammatory cytokine that plays important roles in innate and adaptive immune responses and has been implicated in the pathogenesis of various autoimmune diseases [31]. In cardiovascular disease, IL-15 is found to have increased abundance in atherosclerotic plaques where it promotes T-cell recruitment, activation, and inflammatory responses [32], and elevated IL-15 levels have been associated with cardiovascular organ damage in hypertensive patients [33]. Our analysis demonstrates that IL-15 statistically mediates the relationship between multiple AID categories, suggesting that common inflammatory pathways involving T-cell activation may contribute to cardiovascular risk across diverse AIDs.

For MACE, two more proteins showed consistent mediation across three AID categories: PLAUR/suPAR and TNC. These mediators represent distinct pathophysiological mechanisms. PLAUR/suPAR is expressed on immune cells and endothelial cells, suPAR plays critical roles in inflammation, thrombosis, and tissue remodeling [34]. Elevated suPAR levels have been associated with atherosclerotic CVD [35], incident HF [36], and mortality in population studies [37]. The mechanisms likely involve endothelial dysfunction, enhanced thrombosis and promotion of atherosclerotic plaque instability. TNC is an extracellular matrix glycoprotein with increased abundance during tissue injury and remodeling [38,39]. Elevated TNC has been found to play a role in the pathologic process of MI [40], HF [41,42], and contributes to elevated cardiovascular mortality [43], suggesting that altered extracellular matrix remodeling contributes to acute cardiovascular events in autoimmune conditions.

Beyond the shared mediators, the emergence of NEFL and OMG as mediators specific to neurologic AIDs is notable. NEFL is released into circulation upon axonal injury and is elevated in autoimmune neurologic diseases [44], and has been independently associated with broad range of adverse cardiovascular events and mortality, especially in patients with atrial fibrillation [45,46]. OMG likely reflects ongoing CNS demyelination, which activate innate immune and complement cascades that may contribute to systemic vascular inflammation [47]. Consistent with this, myelin oligodendrocyte glycoprotein antibody-associated disease has been associated with cerebral venous thrombosis and central retinal artery occlusion, suggesting that myelin-targeted autoimmunity may promote thrombo-inflammatory mechanisms contributing to fatal cardiovascular events [48,49]. Both mediators are hypothesis-generating pending further mechanistic validation.

Gene Ontology enrichment analysis of the HIMA-selected consistently implicated cell adhesion and extracellular matrix remodeling, inflammatory cascades, and vascular processes across AID categories, supporting a shared mechanism of endothelial activation and vascular matrix dysregulation linking systemic autoimmune inflammation to MACE. Pathway enrichment *p*-values are nominally significant given the limited number of identified mediators per category, and these results should be interpreted as hypothesis-generating.

### 4.2. Protein Signatures Associated with Lower MACE

Beyond pro-inflammatory mediators, we identified proteins with inverse associations (HR < 1.0) suggesting protective mechanisms across multiple AID categories. IL-18BP is a natural antagonist of IL-18, a pro-inflammatory cytokine involved in atherosclerosis and myocardial injury [50]. IL-18 promotes inflammatory responses through interferon-γ release and enhances atherosclerotic lesion development, while IL-18BP inhibits this process [51]. IL-18BP overexpression prevents fatty streak development and induces stable plaque phenotypes characterized by decreased macrophage content and increased collagen content in experimental atherosclerosis [52]. Elevated serum IL-18 levels predict cardiovascular mortality in patients with stable and unstable angina [53], while IL-18BP has demonstrated protective effects in HF and acute MI [54]. Our findings demonstrate that IL-18BP showed strong protective effects for CV death in GI AIDs (relative importance 17.8%) and MSK AIDs (6.7%), suggesting that elevated IL18BP may reflect compensatory anti-inflammatory mechanisms that counteract IL-18-mediated cardiovascular pathogenesis through plaque stabilization and reduced inflammatory injury in chronic autoimmune conditions.

Our findings extend previous epidemiological studies demonstrating associations between AIDs and CVD [11] by identifying specific molecular mediators underlying these relationships. While prior proteomic study has associated inflammatory proteins with cardiovascular outcomes [55], the mediation-based approach provides statistical evidence consistent with candidate mediating pathways from autoimmune inflammation through specific proteins to cardiovascular events and death. This framework moves beyond correlation by statistically testing mediation effects while controlling for confounders.

## 5. Limitations

Several limitations should be acknowledged. First, while mediation analysis provides evidence for mechanistic pathways, it cannot definitively establish causation. While we adjusted for many confounders including traditional cardiovascular risk factors, residual confounding from unmeasured factors such as diet, physical activity patterns, and socioeconomic status remains possible. The Olink proteomic subcohort contains a slightly lower proportion of AID-free participants than the full cohort, reflecting disease enrichment by the UKB design, introducing potential selection bias. The small number of outcome events in some subcategories renders those mediation findings exploratory, pending replication in larger disease-specific cohorts. Randomized controlled trials targeting identified mediators would be needed to confirm causal roles. Renal function was adjusted for using ICD-10-based chronic kidney disease diagnosis codes rather than continuous eGFR or creatinine/cystatin C measurements. The residual confounding from subclinical renal impairment not captured by a clinical chronic kidney disease diagnosis cannot be excluded, and future studies with available eGFR data should assess the robustness of these mediators after continuous renal function adjustment. The four structural assumptions required for causal mediation inference as detailed in the Section 2 are not fully verifiable in this design—most critically due to unmeasured immunosuppressive therapy use and AID-related organ complications that may create exposure-induced confounders—further supporting a hypothesis-generating rather than causal interpretation. Second, proteomic measurements were obtained at a single baseline time point. While the temporal sequence required for mediation—AID diagnosis preceding protein measurement, which preceded all outcome events—is preserved by design, a single measurement cannot capture fluctuations in protein levels or disease activity over follow-up, and levels at enrollment may not reflect the mediating proteome closest to a given cardiovascular event. Longitudinal proteomic profiling would help clarify these temporal dynamics. Third, the UK Biobank’s well-documented healthy-volunteer bias—with only 5.5% of invitees participating—results in a cohort with healthier lifestyles and lower all-cause mortality than the general UK population. This may apply with force to AID patients, as those with the most severe or active disease may have been less able to participate, potentially underestimating the true AID-MACE associations. Additionally, the cohort has limited racial and ethnic diversity (predominantly white British ancestry), which may further limit generalizability to other populations or patient groups with different genetic backgrounds. Fourth, we did not have detailed data on AIDs activity or treatment plans, which could influence both protein levels and cardiovascular risk. Fifth, proteomic profiling using the Olink platform measures canonical protein products and does not distinguish between proteoforms arising from alternative splicing, post-translational modifications, or genetic variation, which may limit characterization of the proteome complexity underlying these AID-cardiovascular relationships. The Olink panel quantifies approximately 3000 proteins, a fraction of the human proteome; proteins outside this panel could not be evaluated as candidate mediators, and the true mediating pathways between AIDs and MACE may be incompletely captured. In addition, CV death is both a component of MACE and an independent secondary endpoint. Proteins mediating both outcomes cannot be definitively characterized as having broadly composite cardiovascular effects versus mortality-specific effects reflected in both analyses. The secondary CV death analysis should be used as the primary reference for fatal-event-specific mediation, and shared mediators may reflect predominantly mortality-driven mechanisms. Lastly, the HIMA algorithm does not provide confidence intervals for relative importance (proportion mediated) estimates; the relative importance estimates should therefore be interpreted as point estimates only, and their precision is unknown. Additionally, while the protein selection step accounted for the competing risk of non-cardiovascular death using Fine–Gray subdistribution hazard models, the mediation analysis via HIMA relied on standard Cox models that treat competing events as censored, as HIMA does not currently support a competing-risks framework; this inconsistency between the selection and mediation steps could introduce bias. Future proteoform-resolved studies could provide additional mechanistic insight.

## 6. Conclusions

Using high-dimensional mediation analysis in a large population-based cohort, we identified protein mediators linking autoimmune diseases to MACE and CV death through various pathways of cardiac stress, inflammation, thrombosis, and vascular remodeling. We identified two proteins (GDF15 and IL-15) that consistently mediate cardiovascular outcomes across multiple major AID categories, representing universal mediators of immune-related cardiovascular pathogenesis. For MACE specifically, two proteins (PLAUR/suPAR and TNC) showed consistent mediation across these AID categories. Key shared mediators across AID categories and AID-category-specific signatures represent candidate targets for future research into cardiovascular risk stratification and prevention in autoimmune disease patients. These proteins represent readily measurable biomarkers that may warrant evaluation in multi-protein risk-prediction panels, pending replication in independent cohorts and prospective validation. If validated, these findings could inform both universal and AID-specific cardiovascular risk management strategies and help guide future clinical decision-making and therapeutic development for this high-risk population.

## Figures and Tables

**Table 1:** Cohort characteristics.

Variable	Total	No AID	MSK	Vasculitis	GI	Neuro	Rheumatic
(A) Full cohort.							
N	400,633	371,879 (92.8%)	16,568 (4.1%)	680 (0.2%)	8348 (2.1%)	1900 (0.5%)	1258 (0.3%)
Enrollment age (years)	57.0 (50.0–63.0)	57.0 (50.0–63.0)	59.0 (52.0–64.0)	61.0 (55.0–65.0)	58.0 (51.0–63.0)	56.0 (50.0–62.0)	62.0 (57.0–66.0)
Male, n (%)	179,539 (44.8%)	168,461 (45.3%)	6541 (39.5%)	281 (41.3%)	3524 (42.2%)	589 (31.0%)	491 (39.0%)
BMI (kg/m^2^)	27.3 ± 4.7	27.3 ± 4.7	28.1 ± 5.2	28.6 ± 5.5	26.8 ± 4.7	27.0 ± 5.0	27.7 ± 4.9
Ever smoked, n (%)	236,063 (58.9%)	218,781 (58.8%)	10,488 (63.3%)	409 (60.1%)	5100 (61.1%)	1187 (62.5%)	723 (57.5%)
HDL-cholesterol (mg/dL)	54.1 (46.4–65.7)	54.1 (46.4–65.7)	54.1 (46.4–65.7)	54.1 (42.5–65.7)	54.1 (46.4–65.7)	58.0 (46.4–65.7)	54.1 (46.4–65.7)
LDL-cholesterol (mg/dL)	139.2 (116.0–162.4)	139.2 (116.0–162.4)	139.2 (116.0–158.5)	135.3 (108.3–162.4)	131.5 (112.1–154.7)	135.3 (116.0–162.4)	139.2 (116.0–162.4)
Hypertension, n (%)	98,843 (24.7%)	91,062 (24.5%)	5019 (30.3%)	295 (43.4%)	2013 (24.1%)	422 (22.2%)	361 (28.7%)
Type 2 diabetes, n (%)	8397 (2.1%)	7479 (2.0%)	586 (3.5%)	56 (8.2%)	241 (2.9%)	59 (3.1%)	31 (2.5%)
Atrial fibrillation, n (%)	4627 (1.2%)	4168 (1.1%)	275 (1.7%)	22 (3.2%)	119 (1.4%)	22 (1.2%)	54 (4.3%)
Follow-up (years)	14.5 (13.6–15.2)	14.5 (13.6–15.2)	14.4 (13.5–15.2)	14.1 (13.2–15.2)	14.4 (13.5–15.2)	14.4 (13.5–15.2)	14.4 (13.4–15.3)
(B) Proteomic sub-cohort.							
N	43,599	40,090	1999	104	915	349	142
Enrollment age (years)	58.0 (50.0–63.0)	57.0 (50.0–63.0)	59.0 (52.0–64.0)	59.0 (51.0–65.0)	59.0 (52.0–64.0)	56.0 (49.0–62.0)	63.0 (58.2–66.0)
Male, n (%)	19,115 (44.9%)	17,831 (44.5%)	729 (36.5%)	41 (39.4%)	385 (42.1%)	114 (32.7%)	59 (41.5%)
BMI (kg/m^2^)	27.4 ± 4.8	27.4 ± 4.7	28.0 ± 5.2	28.2 ± 5.1	26.9 ± 4.7	27.0 ± 5.1	27.5 ± 5.0
Ever smoked, n (%)	25,127 (59.1%)	23,002 (57.4%)	1272 (63.6%)	64 (61.5%)	577 (63.1%)	222 (63.6%)	83 (58.5%)
HDL-cholesterol (mg/dL)	54.1 (46.4–65.7)	54.1 (46.4–65.7)	54.1 (46.4–65.7)	50.2 (42.5–61.8)	54.1 (46.4–65.7)	54.1 (46.4–65.7)	54.1 (46.4–61.8)
LDL-cholesterol (mg/dL)	135.3 (116.0–158.5)	135.3 (116.0–158.5)	135.3 (112.1–158.5)	131.5 (108.3–162.4)	131.5 (108.3–154.7)	139.2 (116.0–162.4)	131.5 (108.3–158.5)
Hypertension, n (%)	10,898 (25.6%)	9923 (24.8%)	631 (31.6%)	38 (36.5%)	236 (25.8%)	80 (22.9%)	44 (31.0%)
Type 2 diabetes, n (%)	937 (2.2%)	817 (2.0%)	76 (3.8%)	10 (9.6%)	25 (2.7%)	13 (3.7%)	7 (4.9%)
Atrial fibrillation, n (%)	576 (1.4%)	511 (1.3%)	37 (1.9%)	5 (4.8%)	13 (1.4%)	6 (1.7%)	14 (9.9%)
Follow-up (years)	14.4 (13.6–15.2)	14.4 (13.6–15.2)	14.3 (13.4–15.2)	13.9 (13.3–15.1)	14.3 (13.5–15.2)	14.3 (13.4–15.1)	14.3 (13.4–15.3)

Baseline characteristics of UK Biobank participants stratified by autoimmune inflammatory disease (AID) status. Table 1(A) presents the full study cohort (N = 400,633), and Table 1(B) presents the subcohort with available proteomic data for proteome-wide association study (PWAS) and mediation analyses (N = 43,599). Values are presented as mean ± SD for continuous normally distributed variables, median (Q1–Q3) for continuous non-normally distributed variables, or N (%) for categorical variables. AID = autoimmune inflammatory disease; BMI = body mass index; HDL = high-density lipoprotein; LDL = low-density lipoprotein; MSK = musculoskeletal; GI = gastrointestinal; Neuro = neurological.

**Table 2:** Hazard ratios for CV death and composite MACE incidence by autoimmune disease exposures.

	AID Prevalence N (%)	MACE			CV Death		
		Incidence N (%)	sHR (95% CI)	*p*-Value	Incidence N (%)	sHR (95% CI)	*p*-Value
(A) Full cohort.							
No AID	371,879 (92.8%)	35,085 (9.4%)	Reference	N/A	7963 (2.1%)	Reference	N/A
MSK	16,568 (4.1%)	2517 (15.2%)	1.34 (1.28–1.40)	<0.001 *	605 (3.7%)	1.34 (1.23–1.47)	<0.001 *
Vasculitis	680 (0.2%)	280 (41.2%)	1.67 (1.46–1.92)	<0.001 *	83 (12.2%)	1.78 (1.40–2.26)	<0.001 *
GI	8348 (2.1%)	1004 (12.0%)	1.20 (1.12–1.29)	<0.001 *	197 (2.4%)	0.98 (0.84–1.14)	0.77
Neurologic	1900 (0.5%)	238 (12.5%)	1.33 (1.15–1.54)	<0.001 *	39 (2.1%)	0.94 (0.66–1.35)	0.75
Rheumatic fever	1258 (0.3%)	223 (17.7%)	1.38 (1.20–1.60)	<0.001 *	66 (5.2%)	1.51 (1.14–1.99)	0.004 *
(B) Proteomic sub-cohort.							
No AID	40,090 (92.0%)	3472 (8.7%)	Reference	N/A	745 (1.9%)	Reference	N/A
MSK	1999 (4.6%)	370 (18.5%)	1.38 (1.22–1.56)	<0.001	113 (5.7%)	1.41 (1.12–1.79)	0.004 *
Vasculitis	104 (0.2%)	66 (63.5%)	1.71 (1.22–2.38)	0.002	35 (33.7%)	1.98 (1.11–3.54)	0.02 *
GI	915 (2.1%)	133 (14.5%)	1.21 (1.06–1.46)	0.04	41 (4.5%)	1.26 (0.86–1.84)	0.23
Neurologic	349 (0.8%)	36 (10.3%)	0.91 (0.61–1.36)	0.65	5 (1.4%)	0.16 (0.02–1.13)	0.07
Rheumatic fever	142 (0.3%)	34 (23.9%)	1.35 (0.92–1.99)	0.13	13 (9.2%)	1.40 (0.66–3.00)	0.38

Subdistribution hazard ratios (sHR) with 95% confidence intervals (CI) and *p*-values for CV death, and composite MACE incidence in individuals with and without autoimmune disease in the UK Biobank cohort. Fine–Gray models were used to identify associations between AIDs and incident cardiovascular outcomes, treating non-cardiovascular death as competing risks. All models adjusted for age, sex, HDL, LDL, BMI, history of smoking, hypertension, type 2 diabetes, chronic kidney disease, prevalent atrial fibrillation, and prevalent coronary artery disease. MSK: musculoskeletal; GI: gastrointestinal; MACE: major adverse cardiovascular event; CV death: cardiovascular death, BMI, body mass index; HDL, high-density lipoprotein cholesterol; LDL, low-density lipoprotein cholesterol. Asterisks indicate statistical significance (*p* < 0.05).

## Data Availability

The data used in this study are available from the UK Biobank upon reasonable request and are accessible to approved researchers. Access can be requested via the UK Biobank Access Management System at https://www.ukbiobank.ac.uk/ (accessed on 21 December 2025). Data were accessed under UK Biobank application number 34031.

## Abbreviations

The following abbreviations are used in this manuscript:

MACE	Major Adverse Cardiovascular Events
CV death	Cardiovascular Death
AID	Autoimmune Disease
MSK	Musculoskeletal
GI	Gastrointestinal
PWAS	Protein-Wide Association Study
GDF15	Growth Differentiation Factor 15
IL-15	Interleukin-15
PLAUR/suPAR	Plasminogen Activator, Urokinase Receptor

## Appendix A

**Table A1:** ICD-10-CM codes used to select autoimmune diseases and cardiovascular exposures and outcomes.

Condition	ICD-10 Code
Rheumatoid arthritis (sero-positive)	M05
Rheumatoid arthritis (sero-negative)	M06
Psoriatic and enteropathic arthropathies	M07
Systemic lupus erythematosus	M32
Systemic sclerosis (scleroderma)	M34
Sjögren’s syndrome	M35
Systemic connective tissue disorders in diseases classified elsewhere	M36
Ankylosing spondylitis	M45
Other inflammatory spondylopathies	M46
Psoriasis	L40
Other disorders involving the immune mechanism	D89
Polyarteritis nodosa	M30
Other necrotizing vasculopathies	M31
Other disorders of arteries and arterioles	I77
Crohn’s disease	K50
Ulcerative colitis	K51
Diverticular disease of intestine	K57
Intestinal malabsorption	K90
Multiple sclerosis	G35
Other acute disseminated demyelination	G36
Inflammatory polyneuropathy	G61
Myasthenia gravis	G70
Rheumatic fever (without heart involvement)	I00
Essential hypertension	I10
Type 2 diabetes mellitus	E11
Angina Pectoris	I20
Acute myocardial infarction	I21
Subsequent myocardial infarction	I22
Complications following acute myocardial infarction	I23
Other acute ischemic heart diseases	I24
Atrial fibrillation and atrial flutter	I48
Heart failure	I50
Chronic kidney disease	N18
Nontraumatic Subarachnoid Hemorrhage	I60
Ischemic stroke	I63
Stroke, not specified as ischemic or hemorrhagic	I64
Atherosclerosis	I70
Other peripheral vascular diseases	I73

**Table A2:** Significant Protein Mediators of the Association Between Autoimmune Disease and Major Adverse Cardiovascular Events Identified by HIMA.

Protein	Effect of AID on Protein	Effect of Protein on MACE	Mediation Effect		
	Beta (95% CI)	HR (95% CI)	Indirect Effect HR *	Joint *p* **	Proportion Contribution (%)
Musculoskeletal					
ANGPTL3	0.16 (0.12, 0.19)	0.93 (0.89, 0.96)	0.99	6.01 × 10^−4^	2.48
BST2	0.19 (0.15, 0.23)	1.06 (1.02, 1.10)	1.01	2.09 × 10^−4^	2.22
BTN2A1	0.18 (0.14, 0.22)	0.90 (0.86, 0.95)	0.98	2.01 × 10^−6^	3.91
CA6	−0.13 (−0.17, −0.09)	0.93 (0.90, 0.96)	1.01	2.01 × 10^−9^	2.02
CANT1	0.12 (0.07, 0.16)	0.94 (0.90, 0.98)	0.99	4.09 × 10^−3^	1.53
CCL20	0.20 (0.16, 0.24)	0.93 (0.89, 0.96)	0.98	7.11 × 10^−3^	3.31
CD302	0.24 (0.20, 0.28)	1.06 (1.02, 1.11)	1.01	8.43 × 10^−3^	3.09
CNTN1	−0.16 (−0.20, −0.12)	0.93 (0.89, 0.96)	1.01	9.08 × 10^−5^	2.62
CNTN5	−0.11 (−0.15, −0.07)	0.94 (0.91, 0.98)	1.01	1.25 × 10^−3^	1.36
EDA2R	0.16 (0.12, 0.19)	1.10 (1.05, 1.15)	1.01	9.35 × 10^−7^	3.16
EGFR	−0.21 (−0.26, −0.17)	0.88 (0.84, 0.91)	1.03	1.12 × 10^−12^	6.09
ENPP5	−0.12 (−0.16, −0.08)	0.94 (0.91, 0.98)	1.01	1.08 × 10^−3^	1.56
FOLR1	0.19 (0.15, 0.23)	0.92 (0.88, 0.95)	0.98	1.94 × 10^−5^	3.54
FUT3_FUT5	0.12 (0.08, 0.16)	0.95 (0.92, 0.98)	0.99	8.53 × 10^−4^	1.44
GDF15	0.25 (0.21, 0.28)	1.12 (1.07, 1.17)	1.03	9.15 × 10^−9^	5.88
HAVCR1	0.17 (0.13, 0.21)	1.12 (1.08, 1.16)	1.02	7.99 × 10^−12^	4.11
IGFBP7	0.11 (0.07, 0.15)	1.11 (1.06, 1.15)	1.01	4.67 × 10^−7^	2.34
IL15	0.33 (0.29, 0.37)	1.05 (1.02, 1.09)	1.02	1.62 × 10^−5^	3.62
IL1RN	0.15 (0.11, 0.19)	0.93 (0.89, 0.97)	0.99	3.68 × 10^−4^	2.45
ITGA11	−0.17 (−0.21, −0.13)	0.95 (0.91, 0.98)	1.01	2.01 × 10^−5^	1.97
LILRA5	0.21 (0.17, 0.25)	1.09 (1.05, 1.13)	1.02	6.23 × 10^−3^	4.05
LILRB2	0.15 (0.11, 0.19)	0.95 (0.92, 0.98)	0.99	2.51 × 10^−3^	1.64
LTBP2	0.11 (0.07, 0.14)	1.23 (1.18, 1.29)	1.02	8.20 × 10^−9^	4.76
MMP12	0.12 (0.08, 0.16)	1.09 (1.05, 1.12)	1.01	6.87 × 10^−7^	2.03
MSTN	−0.12 (−0.16, −0.08)	0.94 (0.90, 0.97)	1.01	4.65 × 10^−4^	1.69
PLAUR	0.22 (0.18, 0.26)	1.15 (1.10, 1.20)	1.03	4.25 × 10^−8^	6.54
RNASE4	0.11 (0.07, 0.15)	0.94 (0.90, 0.98)	0.99	4.61 × 10^−3^	1.43
SEMA3F	0.16 (0.12, 0.20)	0.94 (0.90, 0.98)	0.99	1.62 × 10^−6^	2.04
TNC	0.16 (0.12, 0.21)	1.06 (1.02, 1.09)	1.01	2.24 × 10^−4^	1.93
TNFRSF11A	0.14 (0.10, 0.18)	0.94 (0.90, 0.98)	0.99	8.36 × 10^−5^	1.73
TNFRSF12A	0.11 (0.07, 0.15)	1.10 (1.05, 1.14)	1.01	1.68 × 10^−4^	2.21
TNFRSF4	0.29 (0.25, 0.33)	1.10 (1.05, 1.15)	1.03	3.07 × 10^−6^	5.92
TNR	−0.12 (−0.16, −0.08)	0.90 (0.87, 0.93)	1.01	1.45 × 10^−8^	2.80
WFDC2	0.19 (0.15, 0.23)	1.07 (1.03, 1.11)	1.01	1.00 × 10^−3^	2.54
Vasculitis					
ADM	0.39 (0.25, 0.52)	1.06 (1.01, 1.10)	1.02	0.0147	2.83
CCL7	0.39 (0.24, 0.54)	1.04 (1.01, 1.08)	1.02	0.0177	2.28
CD302	0.38 (0.23, 0.53)	1.05 (1.01, 1.10)	1.02	8.43 × 10^−3^	2.69
CD59	0.34 (0.20, 0.48)	0.95 (0.91, 0.98)	0.98	4.20 × 10^−3^	2.56
COL18A1	0.37 (0.22, 0.51)	1.06 (1.02, 1.10)	1.02	6.47 × 10^−3^	2.85
EDA2R	0.32 (0.19, 0.45)	1.12 (1.07, 1.17)	1.04	9.35 × 10^−7^	4.96
EPHA1	0.35 (0.20, 0.49)	0.96 (0.92, 1.00)	0.99	0.0325	1.95
FABP4	0.29 (0.17, 0.41)	0.87 (0.83, 0.91)	0.96	2.12 × 10^−6^	5.49
FAP	−0.46 (−0.62, −0.31)	0.91 (0.88, 0.94)	1.05	7.22 × 10^−9^	6.04
GDF15	0.38 (0.25, 0.51)	1.32 (1.26, 1.38)	1.11	9.15 × 10^−9^	13.97
HGF	0.38 (0.24, 0.53)	1.08 (1.04, 1.13)	1.03	2.19 × 10^−4^	4.15
IGFBP2	0.38 (0.24, 0.52)	1.05 (1.01, 1.09)	1.02	0.0172	2.28
IL15	0.45 (0.29, 0.61)	1.07 (1.04, 1.11)	1.03	1.62 × 10^−5^	4.34
LILRA5	0.38 (0.23, 0.53)	1.05 (1.01, 1.09)	1.02	6.23 × 10^−3^	2.58
MMP9	0.41 (0.25, 0.57)	1.05 (1.00, 1.10)	1.02	0.0301	2.83
OSM	0.50 (0.35, 0.66)	0.91 (0.86, 0.96)	0.95	4.66 × 10^−4^	6.25
PLAUR	0.42 (0.27, 0.57)	1.18 (1.13, 1.23)	1.07	4.25 × 10^−8^	9.27
TNC	0.40 (0.24, 0.56)	1.06 (1.03, 1.10)	1.02	2.24 × 10^−4^	3.26
TNFRSF10B	0.35 (0.21, 0.49)	1.11 (1.06, 1.16)	1.04	6.90 × 10^−6^	4.94
TNFRSF11A	0.39 (0.24, 0.53)	0.92 (0.88, 0.96)	0.97	8.36 × 10^−5^	4.28
TNFRSF1A	0.40 (0.26, 0.54)	0.91 (0.86, 0.96)	0.96	5.46 × 10^−4^	5.13
VSIG4	0.43 (0.28, 0.57)	1.09 (1.04, 1.14)	1.04	1.94 × 10^−4^	5.06
Gastrointestinal					
ADM	0.13 (0.08, 0.18)	1.06 (1.02, 1.11)	1.01	0.0147	1.76
AGR2	0.18 (0.12, 0.24)	1.04 (1.01, 1.08)	1.01	0.0122	1.71
ANGPTL3	0.15 (0.09, 0.20)	0.94 (0.90, 0.97)	0.99	6.01 × 10^−4^	2.14
BST2	0.17 (0.10, 0.23)	1.08 (1.03, 1.12)	1.01	2.09 × 10^−4^	2.69
BTN2A1	0.15 (0.09, 0.21)	0.83 (0.79, 0.88)	0.97	2.01 × 10^−6^	5.98
CCL20	0.29 (0.23, 0.35)	0.95 (0.92, 0.99)	0.99	7.11 × 10^−3^	3.20
CD274	0.19 (0.13, 0.25)	1.06 (1.02, 1.10)	1.01	6.68 × 10^−3^	2.44
CD300C	0.15 (0.09, 0.21)	0.94 (0.90, 0.98)	0.99	7.29 × 10^−3^	1.89
CD38	0.13 (0.08, 0.18)	0.94 (0.90, 0.98)	0.99	3.86 × 10^−3^	1.75
CD5	0.16 (0.10, 0.21)	0.95 (0.91, 0.99)	0.99	7.04 × 10^−3^	1.86
CTSO	0.16 (0.10, 0.22)	0.95 (0.91, 0.98)	0.99	3.68 × 10^−3^	2.01
GDF15	0.22 (0.17, 0.28)	1.23 (1.18, 1.29)	1.05	9.15 × 10^−9^	10.54
HAVCR1	0.19 (0.14, 0.25)	1.15 (1.10, 1.19)	1.03	7.99 × 10^−12^	5.92
IGFBP2	0.16 (0.10, 0.21)	1.09 (1.05, 1.13)	1.01	0.0172	3.03
IL15	0.23 (0.17, 0.29)	1.05 (1.02, 1.09)	1.01	1.62 × 10^−5^	2.55
IL6	0.19 (0.13, 0.24)	1.09 (1.05, 1.13)	1.02	5.55 × 10^−6^	3.58
LAMP3	0.15 (0.09, 0.21)	1.06 (1.02, 1.10)	1.01	4.88 × 10^−3^	1.87
MMP10	0.20 (0.14, 0.27)	0.96 (0.93, 0.99)	0.99	0.0127	1.98
MMP12	0.19 (0.13, 0.24)	1.10 (1.06, 1.14)	1.02	6.87 × 10^−7^	3.82
NECTIN2	0.21 (0.15, 0.27)	1.11 (1.06, 1.16)	1.02	2.40 × 10^−6^	5.02
PGF	0.16 (0.11, 0.22)	1.10 (1.06, 1.15)	1.02	1.35 × 10^−5^	3.59
PLAUR	0.15 (0.09, 0.21)	1.20 (1.14, 1.25)	1.03	4.25 × 10^−8^	6.08
RETN	0.15 (0.09, 0.21)	0.94 (0.91, 0.98)	0.99	2.12 × 10^−3^	1.99
SEMA3F	0.14 (0.08, 0.20)	0.89 (0.86, 0.93)	0.98	1.62 × 10^−6^	3.70
TNC	0.22 (0.16, 0.29)	1.08 (1.04, 1.12)	1.02	2.24 × 10^−4^	3.83
TNFRSF11A	0.15 (0.09, 0.21)	0.91 (0.88, 0.95)	0.99	8.36 × 10^−5^	3.04
TNFRSF12A	0.14 (0.08, 0.20)	1.08 (1.04, 1.13)	1.01	1.68 × 10^−4^	2.42
TNFRSF4	0.28 (0.22, 0.34)	1.12 (1.07, 1.18)	1.03	3.07 × 10^−6^	7.28
TNFRSF6B	0.17 (0.11, 0.23)	1.06 (1.02, 1.11)	1.01	1.22 × 10^−3^	2.35
Neurologic					
CA6	−0.29 (−0.38, −0.19)	0.90 (0.87, 0.93)	1.03	2.01 × 10^−9^	13.86
CBLN4	−0.25 (−0.36, −0.15)	0.97 (0.94, 1.01)	1.01	0.114	2.98
ENPP5	−0.32 (−0.42, −0.23)	0.94 (0.91, 0.98)	1.02	1.08 × 10^−3^	8.23
HPGDS	−0.27 (−0.38, −0.17)	0.92 (0.89, 0.95)	1.02	7.89 × 10^−7^	10.16
ITGA11	−0.29 (−0.38, −0.19)	0.92 (0.89, 0.96)	1.02	2.01 × 10^−5^	10.53
ITGAV	−0.39 (−0.49, −0.30)	1.03 (0.99, 1.07)	0.99	0.124	5.35
MSTN	−0.28 (−0.37, −0.18)	0.94 (0.91, 0.97)	1.02	4.65 × 10^−4^	7.46
NEFL	0.29 (0.20, 0.38)	1.23 (1.20, 1.28)	1.06	3.46 × 10^−10^	26.96
OMG	−0.30 (−0.41, −0.20)	0.90 (0.87, 0.93)	1.03	4.39 × 10^−9^	14.47

Significant plasma protein mediators of the association between prevalent autoimmune/inflammatory conditions and major adverse cardiovascular events (MACE). High-dimensional mediation analysis (HIMA) identified mediators with Bonferroni-corrected *p*-value < 0.05. * Indirect effect hazard ratio: beta for the effect of AID on protein × beta (log HR) for the effect of protein on MACE. Confidence interval was not derived by HIMA. ** Joint *p*: defined as the higher of the *p*-values for the AID-protein and protein- MACE path. Protein abbreviations: ADM, Adrenomedullin; AGR2, Anterior Gradient 2; ANGPTL3, Angiopoietin-like Protein 3; BST2, Bone Marrow Stromal Cell Antigen 2; BTN2A1, Butyrophilin Subfamily 2 Member A1; CA6, Carbonic Anhydrase 6; CANT1, Calcium-activated Nucleotidase 1; CBLN4, Cerebellin-4; CCL20, C-C Motif Chemokine Ligand 20; CCL7, C-C Motif Chemokine Ligand 7; CD274, Cluster of Differentiation 274 (PD-L1); CD300C, Cluster of Differentiation 300C; CD302, Cluster of Differentiation 302; CD38, Cluster of Differentiation 38; CD5, Cluster of Differentiation 5; CD59, Cluster of Differentiation 59; CNTN1, Contactin-1; CNTN5, Contactin-5; COL18A1, Collagen Type XVIII Alpha 1 Chain; CTSO, Cathepsin O; EDA2R, Ectodysplasin A2 Receptor; EGFR, Epidermal Growth Factor Receptor; ENPP5, Ectonucleotide Pyrophosphatase/Phosphodiesterase 5; EPHA1, Ephrin Type-A Receptor 1; FABP4, Fatty Acid Binding Protein 4; FAP, Fibroblast Activation Protein Alpha; FOLR1, Folate Receptor 1; FUT3_FUT5, Fucosyltransferase 3/Fucosyltransferase 5; GDF15, Growth Differentiation Factor 15; HAVCR1, Hepatitis A Virus Cellular Receptor 1 (TIM-1); HGF, Hepatocyte Growth Factor; HPGDS, Hematopoietic Prostaglandin D Synthase; IGFBP2, Insulin-like Growth Factor Binding Protein 2; IGFBP7, Insulin-like Growth Factor Binding Protein 7; IL15, Interleukin-15; IL1RN, Interleukin-1 Receptor Antagonist; IL6, Interleukin-6; ITGA11, Integrin Subunit Alpha 11; ITGAV, Integrin Subunit Alpha V; LAMP3, Lysosomal Associated Membrane Protein 3; LILRA5, Leukocyte Immunoglobulin-like Receptor Subfamily A Member 5; LILRB2, Leukocyte Immunoglobulin-like Receptor Subfamily B Member 2; LTBP2, Latent Transforming Growth Factor Beta Binding Protein 2; MMP10, Matrix Metalloproteinase-10; MMP12, Matrix Metalloproteinase-12; MMP9, Matrix Metalloproteinase-9; MSTN, Myostatin; NECTIN2, Nectin Cell Adhesion Molecule 2; NEFL, Neurofilament Light Chain; OMG, Oligodendrocyte Myelin Glycoprotein; OSM, Oncostatin M; PGF, Placental Growth Factor; PLAUR, Plasminogen Activator, Urokinase Receptor; RETN, Resistin; RNASE4, Ribonuclease A Family Member 4; SEMA3F, Semaphorin 3F; TNC, Tenascin C; TNFRSF10B, TNF Receptor Superfamily Member 10B (TRAIL-R2); TNFRSF11A, TNF Receptor Superfamily Member 11A (RANK); TNFRSF12A, TNF Receptor Superfamily Member 12A (Fn14); TNFRSF1A, TNF Receptor Superfamily Member 1A; TNFRSF4, TNF Receptor Superfamily Member 4 (OX40); TNFRSF6B, TNF Receptor Superfamily Member 6B (DcR3); TNR, Tenascin R; VSIG4, V-Set and Immunoglobulin Domain Containing 4; WFDC2, WAP Four-Disulfide Core Domain Protein 2 (HE4).

**Table A3:** Significant Protein Mediators of the Association Between Autoimmune Disease and Cardiovascular Death Identified by HIMA.

Protein	Effect of AID on Protein	Effect of Protein on CV Death	Mediation Effect		
	Beta (95% CI)	HR (95% CI)	Indirect Effect HR *	Joint *p* **	Proportion Contribution (%)
Musculoskeletal					
ANGPT2	0.13 (0.09, 0.17)	1.13 (1.06, 1.21)	1.02	1.03 × 10^−4^	3.64
CNTN1	−0.16 (−0.20, −0.12)	0.83 (0.78, 0.89)	1.03	3.14 × 10^−8^	6.72
EDA2R	0.16 (0.12, 0.19)	1.11 (1.04, 1.20)	1.02	9.01 × 10^−4^	3.95
EGFR	−0.21 (−0.26, −0.17)	0.81 (0.76, 0.87)	1.05	6.55 × 10^−10^	10.27
ENPP5	−0.12 (−0.16, −0.08)	0.88 (0.83, 0.95)	1.02	0.0117	3.49
GDF15	0.25 (0.21, 0.28)	1.16 (1.08, 1.24)	1.04	9.15 × 10^−9^	8.27
HPGDS	−0.15 (−0.20, −0.11)	0.85 (0.80, 0.91)	1.03	1.38 × 10^−7^	5.69
ICAM3	0.12 (0.08, 0.17)	1.10 (1.04, 1.18)	1.01	1.70 × 10^−3^	2.83
IL15	0.33 (0.29, 0.37)	1.11 (1.04, 1.17)	1.03	3.26 × 10^−3^	7.58
IL18BP	0.24 (0.20, 0.28)	0.89 (0.83, 0.95)	0.97	6.40 × 10^−6^	6.66
ITGA11	−0.17 (−0.21, −0.13)	0.89 (0.83, 0.95)	1.02	7.45 × 10^−4^	4.45
LGALS9	0.30 (0.26, 0.34)	0.87 (0.82, 0.94)	0.96	1.52 × 10^−4^	9.24
LTBP2	0.11 (0.07, 0.14)	1.28 (1.20, 1.37)	1.03	8.20 × 10^−9^	6.00
MSTN	−0.12 (−0.16, −0.08)	0.89 (0.83, 0.94)	1.01	1.27 × 10^−3^	3.29
SPON1	0.13 (0.09, 0.17)	1.13 (1.06, 1.20)	1.02	3.49 × 10^−4^	3.62
TFF3	0.11 (0.06, 0.15)	1.12 (1.04, 1.20)	1.01	6.58 × 10^−4^	2.70
TNFRSF10B	0.18 (0.14, 0.22)	1.13 (1.05, 1.22)	1.02	1.50 × 10^−6^	5.24
WFDC2	0.19 (0.15, 0.23)	1.16 (1.09, 1.23)	1.03	4.11 × 10^−6^	6.37
Vasculitis					
ADM	0.39 (0.25, 0.52)	1.10 (1.02, 1.18)	1.04	8.85 × 10^−3^	4.96
AREG	0.44 (0.28, 0.60)	1.09 (1.02, 1.17)	1.04	0.0150	5.04
EDA2R	0.32 (0.19, 0.45)	1.13 (1.05, 1.21)	1.04	9.01 × 10^−4^	5.22
EPHB4	0.38 (0.22, 0.54)	0.86 (0.80, 0.93)	0.95	8.66 × 10^−5^	7.41
GDF15	0.38 (0.25, 0.51)	1.35 (1.25, 1.45)	1.12	9.15 × 10^−9^	15.22
IGFBP2	0.38 (0.24, 0.52)	1.20 (1.13, 1.28)	1.07	1.58 × 10^−7^	9.25
IL15	0.45 (0.29, 0.61)	1.10 (1.03, 1.17)	1.04	3.26 × 10^−3^	5.57
PLAUR	0.42 (0.27, 0.57)	1.19 (1.11, 1.28)	1.07	2.61 × 10^−6^	9.61
TGFA	0.50 (0.34, 0.65)	0.91 (0.84, 0.97)	0.95	7.31 × 10^−3^	6.60
TNFRSF10B	0.35 (0.21, 0.49)	1.25 (1.15, 1.35)	1.08	1.50 × 10^−6^	10.31
TNFRSF11A	0.39 (0.24, 0.53)	0.90 (0.84, 0.97)	0.96	3.01 × 10^−3^	5.40
TNFRSF1A	0.40 (0.26, 0.54)	0.86 (0.80, 0.93)	0.94	2.34 × 10^−4^	7.93
VSIG4	0.43 (0.28, 0.57)	1.14 (1.06, 1.23)	1.06	6.21 × 10^−4^	7.47
Gastrointestinal					
GDF15	0.22 (0.17, 0.28)	1.32 (1.23, 1.42)	1.06	9.15 × 10^−9^	27.48
IGFBP2	0.16 (0.10, 0.21)	1.20 (1.13, 1.28)	1.03	1.58 × 10^−7^	12.72
IL15	0.23 (0.17, 0.29)	1.10 (1.04, 1.17)	1.02	3.26 × 10^−3^	9.77
IL18BP	0.24 (0.18, 0.30)	0.85 (0.79, 0.91)	0.96	6.40 × 10^−6^	17.84
PLAUR	0.15 (0.09, 0.21)	1.20 (1.12, 1.29)	1.03	2.61 × 10^−6^	12.14
TFF3	0.16 (0.10, 0.22)	1.13 (1.05, 1.21)	1.02	6.58 × 10^−4^	8.30
TNFRSF6B	0.17 (0.11, 0.23)	1.17 (1.09, 1.26)	1.03	6.28 × 10^−6^	11.75
Neurologic					
CA6	−0.29 (−0.38, −0.19)	0.88 (0.83, 0.94)	1.04	2.10 × 10^−4^	11.34
CBLN4	−0.25 (−0.36, −0.15)	0.97 (0.92, 1.04)	1.01	0.391	2.18
ENPP5	−0.32 (−0.42, −0.23)	0.92 (0.86, 0.98)	1.03	0.0117	8.70
HPGDS	−0.27 (−0.38, −0.17)	0.82 (0.77, 0.87)	1.06	1.38 × 10^−7^	17.67
ITGA11	−0.29 (−0.38, −0.19)	0.90 (0.84, 0.95)	1.03	7.45 × 10^−4^	10.06
MSTN	−0.28 (−0.37, −0.18)	0.90 (0.85, 0.96)	1.03	1.27 × 10^−3^	9.06
NEFL	0.29 (0.20, 0.38)	1.35 (1.28, 1.44)	1.09	3.46 × 10^−10^	27.94
OMG	−0.30 (−0.41, −0.20)	0.87 (0.82, 0.93)	1.04	3.09 × 10^−5^	13.05

Significant plasma protein mediators of the association between prevalent autoimmune/inflammatory conditions and cardiovascular death (CV death). High-dimensional mediation analysis (HIMA) identified mediators with Bonferroni-corrected *p*-value < 0.05. * Indirect effect hazard ratio: beta for the effect of AID on protein × beta (log HR) for the effect of protein on CV death. Confidence interval was not derived by HIMA. ** Joint *p*: defined as the larger of the *p*-values for the AID-protein and protein- CV death path. Protein abbreviations: ADM, Adrenomedullin; ANGPT2, Angiopoietin-2; AREG, Amphiregulin; CA6, Carbonic Anhydrase 6; CBLN4, Cerebellin-4; CNTN1, Contactin-1; EDA2R, Ectodysplasin A2 Receptor; EGFR, Epidermal Growth Factor Receptor; ENPP5, Ectonucleotide Pyrophosphatase/Phosphodiesterase 5; EPHB4, Ephrin Type-B Receptor 4; GDF15, Growth Differentiation Factor 15; HPGDS, Hematopoietic Prostaglandin D Synthase; ICAM3, Intercellular Adhesion Molecule 3; IGFBP2, Insulin-like Growth Factor Binding Protein 2; IL15, Interleukin-15; IL18BP, Interleukin-18 Binding Protein; ITGA11, Integrin Subunit Alpha 11; LGALS9, Galectin-9; LTBP2, Latent Transforming Growth Factor Beta Binding Protein 2; MSTN, Myostatin; NEFL, Neurofilament Light Chain; OMG, Oligodendrocyte Myelin Glycoprotein; PLAUR, Plasminogen Activator, Urokinase Receptor; SPON1, Spondin-1; TFF3, Trefoil Factor 3; TGFA, Transforming Growth Factor Alpha; TNFRSF10B, TNF Receptor Superfamily Member 10B (TRAIL-R2); TNFRSF11A, TNF Receptor Superfamily Member 11A (RANK); TNFRSF1A, TNF Receptor Superfamily Member 1A; TNFRSF6B, TNF Receptor Superfamily Member 6B (DcR3); VSIG4, V-Set and Immunoglobulin Domain Containing 4; WFDC2, WAP Four-Disulfide Core Domain Protein 2 (HE4).

**Table A4:** Pathway Enrichment for Gene Ontology Using the HIMA-selected Proteins for MACE. Pathways with ≥3 significant proteins and *p* < 0.05 are shown.

GO.ID	Term	Annotated	Significant	Expected	*p*	FDR
MSK						
GO:0098609	cell–cell adhesion	990	10	1.85	5.60 × 10^−5^	0.9
GO:0030334	regulation of cell migration	1078	10	2.01	1.04 × 10^−3^	1
GO:0033209	tumor necrosis factor-mediated signaling pathway	118	3	0.22	1.37 × 10^−3^	1
GO:0045471	response to ethanol	131	3	0.24	1.85 × 10^−3^	1
GO:0046718	symbiont entry into host cell	131	3	0.24	1.85 × 10^−3^	1
GO:0007155	cell adhesion	1551	16	2.89	2.63 × 10^−3^	1
GO:0007411	axon guidance	238	4	0.44	5.63 × 10^−3^	1
GO:0001558	regulation of cell growth	409	6	0.76	6.51 × 10^−3^	1
GO:0007179	transforming growth factor beta receptor signaling pathway	252	4	0.47	6.85 × 10^−3^	1
GO:0007267	cell–cell signaling	1345	9	2.51	7.06 × 10^−3^	1
GO:0043123	positive regulation of canonical NF-kappaB signal transduction	213	3	0.4	7.23 × 10^−3^	1
GO:0007154	cell communication	6739	27	12.57	1.12 × 10^−2^	1
GO:0006629	lipid metabolic process	1393	8	2.6	1.21 × 10^−2^	1
GO:0006493	protein O-linked glycosylation	99	3	0.18	1.31 × 10^−2^	1
GO:0007160	cell–matrix adhesion	240	3	0.45	1.83 × 10^−2^	1
GO:0007611	learning or memory	282	3	0.53	2.09 × 10^−2^	1
GO:0031103	axon regeneration	54	3	0.1	2.44 × 10^−2^	1
GO:0050729	positive regulation of inflammatory response	163	3	0.3	2.49 × 10^−2^	1
GO:0043410	positive regulation of MAPK cascade	464	5	0.87	2.85 × 10^−2^	1
GO:0009612	response to mechanical stimulus	223	3	0.42	3.00 × 10^−2^	1
GO:0070665	positive regulation of leukocyte proliferation	168	4	0.31	3.05 × 10^−2^	1
GO:0050867	positive regulation of cell activation	392	5	0.73	3.32 × 10^−2^	1
GO:0050730	regulation of peptidyl-tyrosine phosphorylation	156	3	0.29	4.00 × 10^−2^	1
GO:0030335	positive regulation of cell migration	600	5	1.12	4.44 × 10^−2^	1
GO:0006955	immune response	2172	12	4.05	4.48 × 10^−2^	1
GO:0001503	ossification	458	4	0.85	4.76 × 10^−2^	1
GI						
GO:0042102	positive regulation of T cell proliferation	105	4	0.16	2.10 × 10^−4^	1
GO:0007267	cell–cell signaling	1345	9	2.08	2.90 × 10^−4^	1
GO:0009612	response to mechanical stimulus	223	4	0.34	3.70 × 10^−4^	1
GO:0070555	response to interleukin-1	116	3	0.18	8.00 × 10^−4^	1
GO:0051384	response to glucocorticoid	136	3	0.21	1.19 × 10^−3^	1
GO:0032526	response to retinoic acid	106	3	0.16	1.55 × 10^−3^	1
GO:0022617	extracellular matrix disassembly	65	3	0.1	2.23 × 10^−3^	1
GO:0008284	positive regulation of cell population proliferation	991	12	1.53	2.87 × 10^−3^	1
GO:0007565	female pregnancy	190	3	0.29	3.08 × 10^−3^	1
GO:0097190	apoptotic signaling pathway	632	4	0.98	3.11 × 10^−3^	1
GO:0006954	inflammatory response	927	7	1.43	1.10 × 10^−2^	1
GO:0001649	osteoblast differentiation	262	3	0.41	1.10 × 10^−2^	1
GO:0046718	symbiont entry into host cell	131	3	0.2	1.17 × 10^−2^	1
GO:0001666	response to hypoxia	316	3	0.49	1.25 × 10^−2^	1
GO:0032868	response to insulin	324	3	0.5	1.34 × 10^−2^	1
GO:0032496	response to lipopolysaccharide	357	4	0.55	1.42 × 10^−2^	1
GO:1903900	regulation of viral life cycle	115	3	0.18	1.44 × 10^−2^	1
GO:0045088	regulation of innate immune response	482	4	0.75	1.83 × 10^−2^	1
GO:0070665	positive regulation of leukocyte proliferation	168	7	0.26	2.20 × 10^−2^	1
GO:0030335	positive regulation of cell migration	600	6	0.93	2.39 × 10^−2^	1
GO:0042113	B cell activation	286	4	0.44	2.91 × 10^−2^	1
GO:0007165	signal transduction	6207	23	9.6	2.99 × 10^−2^	1
GO:0010628	positive regulation of gene expression	1234	8	1.91	3.37 × 10^−2^	1
GO:0042098	T cell proliferation	217	5	0.34	3.71 × 10^−2^	1
GO:0043410	positive regulation of MAPK cascade	464	3	0.72	3.96 × 10^−2^	1
GO:0034097	response to cytokine	1016	9	1.57	4.17 × 10^−2^	1
GO:0009725	response to hormone	961	9	1.49	4.72 × 10^−2^	1
Vasculitis						
GO:0050729	positive regulation of inflammatory response	163	6	0.19	1.10 × 10^−5^	0.1
GO:0043410	positive regulation of MAPK cascade	464	6	0.54	1.90 × 10^−5^	0.1
GO:0009612	response to mechanical stimulus	223	5	0.26	5.10 × 10^−4^	0.9
GO:0008284	positive regulation of cell population proliferation	991	8	1.16	7.00 × 10^−4^	1
GO:0033209	tumor necrosis factor-mediated signaling pathway	118	3	0.14	1.31 × 10^−3^	1
GO:0051897	positive regulation of phosphatidylinositol 3-kinase/protein kinase B signal transduction	190	3	0.22	1.37 × 10^−3^	1
GO:0043123	positive regulation of canonical NF-kappaB signal transduction	213	3	0.25	1.90 × 10^−3^	1
GO:0032868	response to insulin	324	3	0.38	2.37 × 10^−3^	1
GO:0001934	positive regulation of protein phosphorylation	414	7	0.49	3.06 × 10^−3^	1
GO:0001649	osteoblast differentiation	262	3	0.31	6.29 × 10^−3^	1
GO:0045471	response to ethanol	131	3	0.15	7.38 × 10^−3^	1
GO:0032496	response to lipopolysaccharide	357	3	0.42	8.72 × 10^−3^	1
GO:0007565	female pregnancy	190	3	0.22	8.97 × 10^−3^	1
GO:0007155	cell adhesion	1551	8	1.82	1.77 × 10^−2^	1
GO:0045834	positive regulation of lipid metabolic process	133	3	0.16	1.81 × 10^−2^	1
GO:0043065	positive regulation of apoptotic process	568	5	0.67	1.93 × 10^−2^	1
GO:0050867	positive regulation of cell activation	392	3	0.46	2.15 × 10^−2^	1
GO:0008625	extrinsic apoptotic signaling pathway via death domain receptors	86	3	0.1	2.95 × 10^−2^	1
GO:0006954	inflammatory response	927	11	1.09	3.19 × 10^−2^	1
GO:0001525	angiogenesis	640	4	0.75	3.59 × 10^−2^	1
GO:0008285	negative regulation of cell population proliferation	794	5	0.93	3.60 × 10^−2^	1
GO:0018108	peptidyl-tyrosine phosphorylation	198	4	0.23	3.91 × 10^−2^	1
GO:0007267	cell–cell signaling	1345	7	1.58	4.73 × 10^−2^	1
Neuro						
GO:0007154	cell communication	6739	7	3.23	8.44 × 10^−3^	1
GO:0040011	locomotion	1370	3	0.66	7.62 × 10^−3^	1

**Table A5:** Pathway Enrichment for Gene Ontology Using the HIMA-selected Proteins for CV death. Pathways with ≥3 significant proteins and *p* < 0.05 are shown.

GO.ID	Term	Annotated	Significant	Expected	*p*	FDR
MSK						
GO:0001525	angiogenesis	640	8	1.26	0.00 × 10^0^	1
GO:0033627	cell adhesion mediated by integrin	89	3	0.18	1.00 × 10^−3^	1
GO:0007229	integrin-mediated signaling pathway	111	3	0.22	1.00 × 10^−3^	1
GO:0007160	cell–matrix adhesion	240	4	0.47	1.00 × 10^−3^	1
GO:0032496	response to lipopolysaccharide	357	4	0.7	2.00 × 10^−3^	1
GO:0098609	cell–cell adhesion	990	10	1.95	2.00 × 10^−3^	1
GO:0007155	cell adhesion	1551	17	3.06	3.00 × 10^−3^	1
GO:0048659	smooth muscle cell proliferation	178	3	0.35	4.00 × 10^−3^	1
GO:0043123	positive regulation of canonical NF-kappaB signal transduction	213	3	0.42	8.00 × 10^−3^	1
GO:0007267	cell–cell signaling	1345	9	2.65	9.00 × 10^−3^	1
GO:0008217	regulation of blood pressure	194	3	0.38	1.40 × 10^−2^	1
GO:0071356	cellular response to tumor necrosis factor	219	4	0.43	1.50 × 10^−2^	1
GO:0030198	extracellular matrix organization	338	4	0.67	1.50 × 10^−2^	1
GO:0007596	blood coagulation	241	3	0.48	1.60 × 10^−2^	1
GO:0010467	gene expression	7384	15	14.56	2.90 × 10^−2^	1
GO:0040011	locomotion	1370	9	2.7	3.10 × 10^−2^	1
GO:0048812	neuron projection morphogenesis	661	5	1.3	3.40 × 10^−2^	1
GO:0006508	proteolysis	1552	7	3.06	3.90 × 10^−2^	1
GO:0045766	positive regulation of angiogenesis	192	3	0.38	3.90 × 10^−2^	1
GI						
GO:0032526	response to retinoic acid	106	3	0.12	1.00 × 10^−3^	1
GO:0007565	female pregnancy	190	3	0.21	1.00 × 10^−3^	1
GO:0061041	regulation of wound healing	140	3	0.16	5.00 × 10^−3^	1
GO:0007155	cell adhesion	1551	9	1.74	1.40 × 10^−2^	1
GO:0008284	positive regulation of cell population proliferation	991	7	1.11	1.80 × 10^−2^	1
GO:0007267	cell–cell signaling	1345	5	1.51	2.40 × 10^−2^	1
GO:0030155	regulation of cell adhesion	819	7	0.92	3.30 × 10^−2^	1
GO:0010628	positive regulation of gene expression	1234	5	1.38	3.70 × 10^−2^	1
GO:0022409	positive regulation of cell–cell adhesion	337	4	0.38	3.70 × 10^−2^	1
Vasculitis						
GO:0038065	collagen-activated signaling pathway	62	3	0.06	4.00 × 10^−3^	1
GO:0048009	insulin-like growth factor receptor signaling pathway	102	3	0.1	2.00 × 10^−3^	1
GO:0007160	cell–matrix adhesion	240	3	0.24	5.00 × 10^−3^	1
GO:0045785	positive regulation of cell adhesion	499	3	0.51	4.80 × 10^−2^	1
GO:0030335	positive regulation of cell migration	600	3	0.61	2.20 × 10^−2^	1
GO:0098609	cell–cell adhesion	990	5	1	6.00 × 10^−3^	1
Neuro						
GO:0033627	cell adhesion mediated by integrin	89	4	0.04	0.00 × 10^0^	0.2
GO:0007229	integrin-mediated signaling pathway	111	3	0.05	0.00 × 10^0^	0.4
GO:0007160	cell–matrix adhesion	240	3	0.1	0.00 × 10^0^	0.2
GO:0098742	cell–cell adhesion via plasma-membrane adhesion molecules	280	3	0.12	1.00 × 10^−3^	0.6
GO:0040011	locomotion	1370	4	0.58	5.00 × 10^−3^	1
GO:0098609	cell–cell adhesion	990	4	0.42	4.70 × 10^−2^	1
GO:0007155	cell adhesion	1551	5	0.66	5.00 × 10^−2^	1
